# Postoperative ratio of C-reactive protein to albumin is an independent prognostic factor for gastric cancer

**DOI:** 10.1186/s40001-023-01334-w

**Published:** 2023-09-21

**Authors:** Chenxi Li, Xuhui Yang, Hui Li, Yan Fu, Wenying Wang, Xin Jin, Lihua Bian, Liang Peng

**Affiliations:** 1 Senior Department of Oncology, The Fifth Medical Center of PLA General Hospital, Beijing, China; 2 Department of Oncology, The Fourth Medical Center of PLA General Hospital, Beijing, China; 3 Senior Department of Obstetrics & Gynecology, The Seventh Medical Center of PLA General Hospital, Beijing, China; 4 Senior Department of Hepato-Pancreato-Biliary Surgery, The First Medical Center of PLA General Hospital, Beijing, China; 5 Department of Obstetrics and Gynecology, Hainan Hospital of PLA General Hospital, Sanya, China

**Keywords:** Preoperative CRP/Alb ratio, Postoperative CRP/Alb ratio, Gastric cancer, Prognosis, Overall survival

## Abstract

**Objective:**

The role of postoperative of the ratio of c-reactive protein to albumin (CRP/Alb ratio) in the prognosis of gastric cancer is rarely evaluated. Our purpose was to investigate the correlation of the postoperative CRP/Alb ratio and long-term prognosis of gastric cancer.

**Methods:**

We enrolled 430 patients who suffered from radical gastrectomy. The commonly used inflammatory indices, clinical-pathological characteristics and oncologic outcomes were recorded. The median was used to the cut-off value for preoperative and postoperative CRP/Alb ratio, respectively. Kaplan–Meier analysis and Cox proportional hazards regression model were performed to determine its prognostic significance.

**Results:**

In univariate analysis, there were significant differences were observed in overall survival (OS) according to perioperative CRP/Alb ratio, c-reactive protein (CRP), serum albumin (Alb), respectively. According to the multivariate analysis, higher postoperative CRP/Alb ratio (HR 2.03, 95% CI 1.55–2.66, P < 0.001), lower postoperative albumin (Alb), higher preoperative c-reactive protein (CRP) and higher postoperative CRP were indicated a shorter overall survival.

**Conclusion:**

Postoperative inflammatory factors in patients with gastric cancer should be pay attention, especially postoperative CRP/Alb ratio may be an independent predictor of long-term prognosis of gastric cancer.

## Introduction

In 2020, there were 19.29 million new cancer cases worldwide, of which 4.57 million were in China [1]. Gastric cancer ranks third in the number of new cancer cases and cancer-related deaths respectively [2], making it the one of the major cancer burdens in China [1]. Due to the insidious onset of gastric cancer, most patients were diagnosed in the middle or advanced stages. In the past few decades, although the survival of gastric cancer patients has been prolonged with the continuous updating of surgical techniques and chemoradiotherapy, the prognosis of gastric cancer is still unsatisfactory. Therefore, it is of great significance to identify prognostic factors, so as to guide the individualized therapy of gastric cancer patients.

A number of studies have demonstrated that malnutrition, serum lipids and the systemic inflammatory response may play important roles in cancer prognosis and carcinogenesis. We previously reported that postoperative high-density lipoprotein cholesterol might be an independent prognostic factor of gastric cancer [3]. Also, the commonly used inflammatory indices including the Alb, albumin/globulin ratio, CRP, neutrophils, neutrophils-platelet score, the CRP/Alb ratio, GPS, mGPS, HS-GPS, fibrinogen-to-albumin ratio, monocyte-to-lymphocyte ratio, lymphocyte to monocyte ratio, and neutrophil percentage-to-albumin ratio have been reported to have prognostic value in patients with gastric cancer, colorectal cancer, hepatocellular carcinoma, bladder cancer and so on [4–16]. Clinically, CRP is used mainly as a marker for inflammation. Inflammatory responses have been thought of as antitumor, but cancer patients are often defective in inflammatory responses. Alb are used to assess the nutritional status and severity of disease for a cancer patient. Previous studies have found that high pretreatment CRP/Alb ratio is associated with poor prognosis in esophageal cancer, gastric cancer, ovarian cancer, pancreatic cancer, hepatocellular carcinoma and anal squamous cell carcinoma [17–23] In addition, one study report that postoperative CRP/Alb ratio was an independent prognostic factor for surgical complications and had a higher diagnostic accuracy than CRP alone of gastric cancer [24]. However, the role of postoperative of CRP/Alb ratio in the prognosis of gastric cancer is rarely evaluated.

Therefore, we aimed to investigate the correlation of the several markers of systemic inflammatory response and prognosis of gastric cancer. In particular, whether postoperative CRP/Alb ratio can predict the long-term prognosis of gastric cancer.

## Materials/methods

### Patients

We identified patients who received gastrectomy with curative (R0) resection at PLA general hospital between January 2011 and December 2013. Before surgery, tissues were obtained by endoscopy in all patients, and the pathological results were confirmed gastric cancer. Patients were followed up every two years. Death or loss of follow-up was the endpoint, the overall survival (OS) was defined as the period from histological confirmation of neoplasia to the endpoint. The disease-free survival (DFS) was the period from surgery to the cancer recurrence or metastasis. All patients were followed up from the day of surgery to January 1, 2021. Patients who had serious cardiovascular and cerebrovascular diseases, loss of follow-up, overall survival of less than 6 months, and incomplete information were excluded. All patients provided written informed consent. The Medical Ethics Committee of PLA General Hospital has reviewed and approved this project, and this were followed in accordance with relevant laws, Declaration of Helsinki and other ethical principles (S2022-262-02). All participants have signed informed consent forms.

### Data collection

The retrospective data include the following: age; gender; tumor location; smoke; history of family; degree of pathological differentiation, histological type; tumor node metastasis (TNM) classification and chemotherapy regimen.

Blood samples were measured in early morning within 1 week before surgery and 1 month after surgery. We used standard value given by Cobas c 701 chemistry analyzer (Roche) as cut-off to define the reference values of CRP and Alb. The low and high reference values were 35 g/L for Alb, 10 mg/L for CRP. The definition of CRP/Alb ratio is CRP divided by the albumin.

### Statistical analysis

The SPSS version 22.0 software was used for statistical analysis in this study. The clinicopathologic characteristics and inflammatory indices were all count data, which presented as the percentage (%), and Pearson’s chi-square test was used to compare each group. The median was used to determine the cut-off value for preoperative and postoperative CRP/Alb ratio, respectively. A survival analysis was performed using Kaplan Meier’s method, and prognostic factors were investigated in a multivariable analysis using a Cox proportional hazards regression model. *P* value < 0.05 was considered statistically significant.

## Results

430 patients were enrolled in this study, and the median follow-up time was 107.1 ± 10.8 months (range 85.4–134 months). Table 1 shows the basic clinical data of 430 patients with gastric cancer. The majority of patients were men, accounting for 77 percent of the total. 280 (65.1%) patients were younger than 60 years old. Postoperative pathological results showed that 255 (59.3%) cases were adenocarcinoma, 21(4.9%) cases were signet ring cell carcinoma, and the remaining 154 (35.8%) cases were mixed type. Most of the tumor tissues were poorly differentiated, with 361 (84%) cases of low differentiation, 57 (13.3%) cases of moderate differentiation, and only 12 (2.8%) cases of high differentiation. The number of TNM classification of patients in this study was as follows: 33 patients in stage I, 120 patients in stage II, 277 patients in stage III. Most of the subjects had no history of smoking and no family history of malignancy. The median was used to the cut-off value for preoperative and postoperative CRP/Alb ratio, respectively. The cutoff value is 0.08 for preoperative CRP/Alb ratio and 0.13 for postoperative CRP/Alb ratio.

**Table 1:** Characteristics of the 430 gastric cancer patients

Characteristics	Patients	%
Age (year)		
< 60	280	65.1
≥ 60	150	34.9
Sex		
Male	331	77.0
Female	99	23.0
Tumor location		
Lower third	155	36.0
Middle third	120	27.9
Upper third	123	28.6
Diffuse	32	7.4
Differentiation		
High	12	2.8
Moderate	57	13.3
Low	361	84.0
T stage		
1	31	7.2
2	47	10.9
3	70	16.3
4	282	65.6
N stage		
0	96	22.3
1	86	20.0
2	102	23.7
3a	92	21.4
3b	54	12.6
TNM stage		
I	33	7.7
II	120	27.9
III	277	64.4
Histological type		
Adenocarcinoma	255	59.3
Signet ring cell	21	4.9
Mixed	154	35.8
Family history		
Yes	112	26.0
No	318	74.0
Smoke		
Yes	94	21.9
No	336	78.1

Table 2 shows the characteristics of various inflammatory indicators before and after surgery. According to the cut-off value for CRP/Alb ratio, patients were divided into low group and high group. Before surgery, most of the patients' Alb and CRP are within the normal range. However, the proportion of postoperative abnormal inflammatory indicators showed an increasing trend.

**Table 2:** Characteristics of preoperative and postoperative inflammatory indicators

	Preoperative		Postoperative	
	n	%	n	%
Alb (g/L)				
< 35	28	6.5	70	16.3
≥ 35	402	93.5	360	83.7
CRP (mg/L)				
≤ 10	378	87.9	350	81.4
> 10	52	12.1	80	18.6
CRP/Alb ratio				
Low	213	49.5	215	50.0
High	217	50.5	215	50.0

The 3-year and 5-year DFS rates were 60.6% and 43.3%, respectively. In univariate analyses of DFS (Table 3, left panel), the factors associated with the gastric cancer DFS were the sex, differentiation, T stage, N stage, TNM stage, family history, smoke history, preoperative CRP/Alb ratio, postoperative CRP/Alb ratio, preoperative Alb, preoperative CRP, postoperative Alb and postoperative CRP. To control confounding factors, multivariate regression analysis was used. As shown in Table 3 (right panel), Poor tumor differentiation, late T stage, late N stage, history of smoking, higher postoperative CRP, lower postoperative Alb, higher preoperative CRP/Alb ratio (HR 1.42, 95% CI 1.08–1.87, P = 0.012) and higher postoperative CRP/Alb ratio (HR 1.72, 95% CI 1.29–2.30, P < 0.001) were associated with poor DFS.

**Table 3:** Univariate and multivariate cox proportional hazard model of gastric cancer with DFS

	Univariate analysis		Multivariate analysis	
	HR (95% CI)	P-value	HR (95% CI)	*P*-value
Age (year)				
< 60	1	0.226		
≥ 60	1.13 (0.91–1.39)			
Sex				
Male	1	0.006		
Female	0.71 (0.56–0.91)			
Tumor location				
Upper third	1	0.195		
Middle third	0.97 (0.74–1.26)	0.796		
Lower third	0.82 (0.64–1.05)	0.112		
Diffuse	1.19 (0.79–1.80)	0.413		
Differentiation				
Well	1	0.007	1	0.018
Moderate	3.71 (1.65–8.38)	0.002	3.26 (1.42–7.48)	0.005
Poor	3.53 (1.51–8.28)	0.004	3.48 (1.45–8.33)	0.005
T stage				
1	1	0.000	1	0.000
2	1.91 (1.03–3.55)	0.041	2.10 (1.12–4.00)	0.021
3	4.29 (2.39–7.69)	0.000	4.55 (2.49–8.31)	0.000
4	5.40 (3.14–9.28)	0.000	4.96 (2.82–8.73)	0.000
N stage				
0	1	0.000	1	0.000
1	1.72 (1.25–2.37)	0.001	2.08 (1.48–2.90)	0.000
2	2.35 (1.74–3.19)	0.000	1.93 (1.40–2.65)	0.000
3a	2.78 (2.03–3.80)	0.000	2.14 (1.55–2.95)	0.000
3b	5.47 (3.80–7.86)	0.000	3.42 (2.32–5.06)	0.000
TNM stage				
I + II	1	0.000		
III	3.50 (2.78–4.39)			
Histological type				
Adenocarcinoma	1	0.833		
Signet ring cell	0.92 (0.57–1.49)	0.733		
Mixed	1.05 (0.85–1.29)	0.664		
Family history				
Yes	1	0.006		
No	0.72 (0.57–0.91)			
Smoke				
No	1	0.001	1	0.005
Yes	1.52(1.20–1.92)		1.43 (1.11–1.84)	
Pre CRP/Alb ratio				
< 0.08	1	0.000	1	0.012
≥ 0.08	3.03 (2.45–3.74)		1.42 (1.08–1.87)	
Post CRP/Alb ratio				
< 0.13	1	0.000	1	0.000
≥ 0.13	3.52 (2.84–4.37)		1.72 (1.29–2.30)	
Pre Alb (g/L)				
< 35	1	0.000		
≥ 35	0.25 (0.17–0.38)			
Post Alb (g/L)				
< 35	1	0.000	1	0.000
≥ 35	0.21 (0.16–0.27)		0.47 (0.34–0.65)	
Pre CRP (mg/L)				
≤ 10	1	0.000		
> 10	5.91 (4.25–8.21)			
Post CRP (mg/L)				
≤ 10	1	0.000	1	0.000
> 10	5.42 (4.10–7.17)		2.11 (1.49–3.00)	

**Fig. 1:**
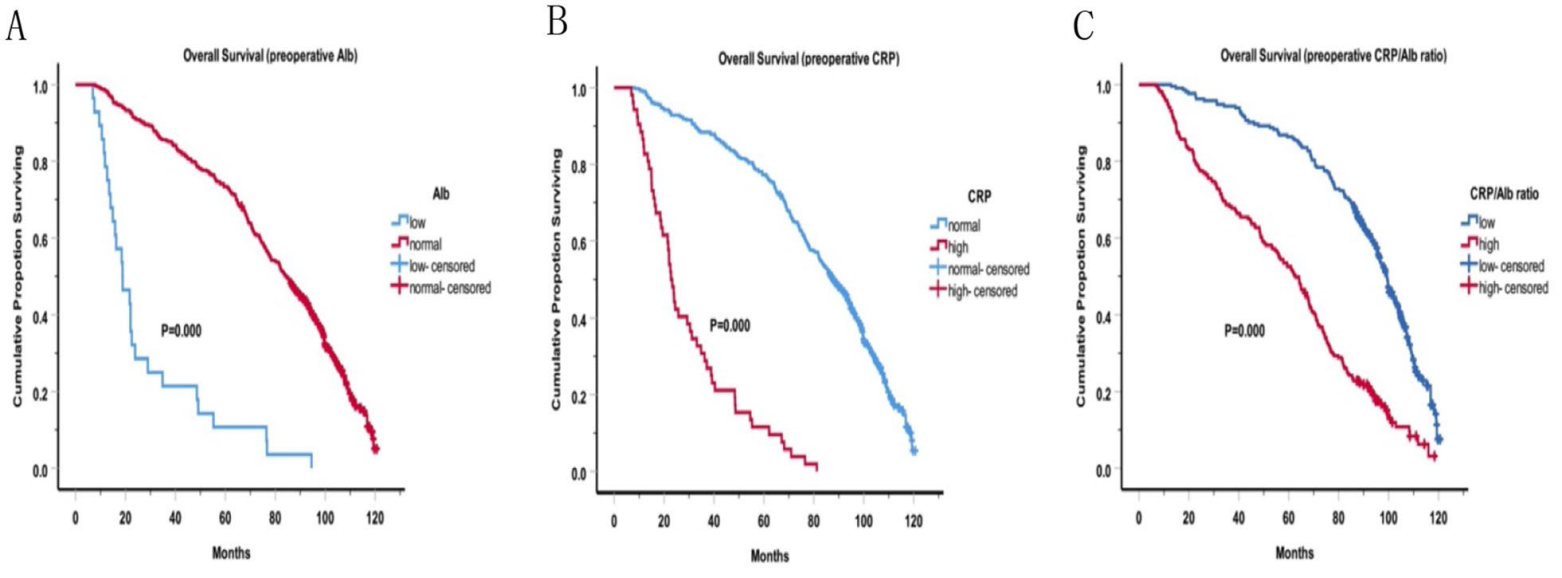
Overall survival based on preoperative inflammatory indicators. Overall survival based on preoperative CRP, albumin and CRP/Alb ratio in patients with gastric cancer, respectively

**Fig. 2:**
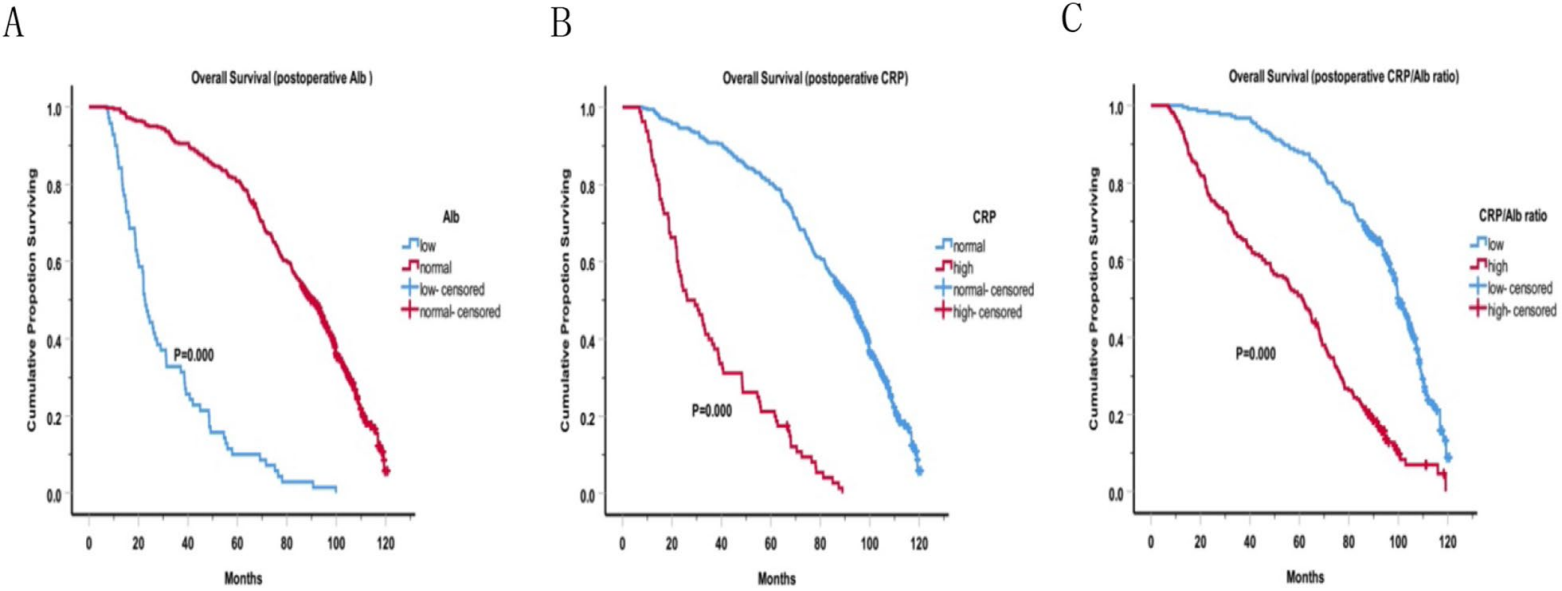
Overall survival based on postoperative inflammatory indicators. Overall survival based on postoperative CRP, albumin and CRP/Alb ratio in patients with gastric cancer, respectively

The median OS for all patients is 80 months. The OS curves are shown in the figures. Before surgery, we found that patients with higher CRP levels, lower albumin levels and higher CRP/Alb ratio had shorter survival, as shown in Fig. 1A–C. Similarly, patients with higher CRP levels (median OS: 26.1 months vs. 92.5 months, P < 0.001), lower albumin levels (median OS: 22.5 months vs. 89.7 months, P < 0.001) and higher CRP/Alb ratio (median OS: 61.1 months vs. 99.9 months, P < 0.001) after surgery had shorter survival, as shown in Fig. 2A–C. Furthermore, Cox proportional hazard model was used to analyze the relationship between Clinicopathologic features and OS. In the univariate analysis, the sex (P = 0.029), diffuse tumor location (P = 0.01), poorly differentiation (P = 0.049), advanced T stage (P = 0.000), advanced N stage (P = 0.000), advanced TNM stage (P = 0.000), family history (P = 0.039), smoke history (P = 0.005), lower preoperative Alb (P = 0.000), lower postoperative Alb (P = 0.000), higher preoperative CRP (P = 0.000), higher postoperative CRP (P  = 0.000), higher preoperative CRP/Alb ratio (HR 2.91, 95% CI 2.32–3.66, P = 0.000) and higher postoperative CRP/Alb ratio (HR 3.50, 95% CI 2.77–4.41, P = 0.000) were prognostic factors for poor OS (Table 4 left panel). According to the multivariate analysis, the results are presented in Table 4 (right panel), increasing T stage (P = 0.001), lymph node stage (P = 0.001), poor differentiation(P = 0.005), mixed histological type (P = 0.051), lower postoperative Alb (P = 0.000), higher preoperative CRP (P = 0.048), higher postoperative CRP (P = 0.001) and higher postoperative CRP/Alb ratio (HR 2.03, 95% CI 1.55–2.66, P = 0.000) were all indicated a shorter overall survival.

**Table 4:** Univariate and multivariate cox proportional hazard model of gastric cancer with OS

	Univariate analysis		Multivariate analysis	
	HR (95% CI)	P-value	HR (95% CI)	*P*-value
Age (year)				
< 60	1	0.849		
≥ 60	1.02 (0.81–1.26)			
Sex				
Male	1	0.029		
Female	0.74 (0.56–0.97)			
Tumor location				
Upper third	1	0.010		
Middle third	1.05 (0.79–1.41)	0.736		
Lower third	0.89 (0.68–1.17)	0.397		
Diffuse	1.82 (1.19–2.79)	0.006		
Differentiation				
Well	1	0.049	1	0.005
Moderate	2.66 (1.18–6.02)	0.019	1.42 (0.61–3.27)	0.417
Poor	2.93 (1.24–6.91)	0.014	2.40 (0.99–5.79)	0.052
T stage				
1	1	0.000	1	0.000
2	2.21 (1.04–4.70)	0.040	2.30 (1.07–4.94)	0.033
3	3.09 (1.47–6.44)	0.003	2.76 (1.30–5.87)	0.008
4	5.97 (3.07–11.64)	0.000	5.32 (2.67–10.60)	0.000
N stage				
0	1	0.000	1	0.000
1	2.08 (1.45–2.99)	0.000	2.50 (1.69–3.70)	0.000
2	2.49 (1.76–3.52)	0.000	2.35 (1.61–3.42)	0.000
3a	3.11 (2.18–4.44)	0.000	2.71 (1.85–3.96)	0.000
3b	6.00 (4.05–8.90)	0.000	4.71 (3.06–7.26)	0.000
TNM stage				
I + II	1	0.000		
III	3.91 (3.01–5.08)			
Histological type				
Adenocarcinoma	1	0.456	1	0.051
Signet ring cell	1.31 (0.80–2.15)	0.287	1.39 (0.83–2.33)	0.210
Mixed	1.10 (0.88–1.39)	0.403	1.33 (1.04–1.71)	0.022
Family history				
Yes	1	0.039		
No	0.77 (0.60–0.99)			
Smoke				
No	1	0.005		
Yes	1.44 (1.12–1.85)			
Pre CRP/Alb ratio				
< 0.08	1	0.000		
≥ 0.08	2.91 (2.32–3.66)			
Post CRP/Alb ratio				
< 0.13	1	0.000	1	0.000
≥ 0.13	3.50 (2.77–4.41)		2.03 (1.55–2.66)	
Pre Alb (g/L)				
< 35	1	0.000		
≥ 35	0.15 (0.10–0.22)			
Post Alb (g/L)				
< 35	1	0.000	1	
≥ 35	0.12 (0.09–0.17)		0.34 (0.24–0.50)	
Pre CRP (mg/L)				
≤ 10	1	0.000		0.048
> 10	8.63 (6.17–12.08)		1.61 (1.01–2.59)	
Post CRP (mg/L)				
≤ 10	1	0.000	1	0.001
> 10	7.02 (5.27–9.35)		2.15 (1.37–3.39)	

## Discussion

The inflammatory response is a powerful tumor promoter and is also thought to have anti-tumor effects, but the majority of tumor patients have defects in the inflammatory response. Basic and clinical studies have shown that inflammation and nutritional status play important roles in the cancer growth, cancer progression and cancer prognosis [25–29]. In recent years, there have been many researches on the relationship between pre-treatment inflammatory indicators and tumor prognosis, such as CRP, neutrophils-platelet score, the CRP/Alb ratio, GPS, mGPS, HS-GPS and so on. Although studies have reported the prognostic value of these indicators, this is the first study to estimate the postoperative inflammatory indices level, especially CRP/Alb ratio on OS for early and locally advanced gastric cancer.

CRP is a nonspecific inflammatory marker produced by the liver. It may increase in patients with malignant tumors, for example, the combined detection of CRP and AFP were used in the differential diagnosis of hepatocellular carcinoma and benign liver diseases, and in the judgment of the efficacy and prognosis of liver cancer. It can also predict the prognosis of various tumors [30–32]. Serum albumin is synthesized in the liver. The nutritional state of the body, hormone balance, plasma osmotic pressure and other factors can affect its synthesis. Low serum albumin level may predict short survival in tumor patients. In this study, using Kaplan–Meier to assess, and the results were consistent with previous studies: preoperative high CRP level or low albumin level were both indicators of poor prognosis in patients with gastric cancer. Meanwhile, we also analyzed the relationship between postoperative CRP or albumin and the long-term survival of patients with gastric cancer, and we also found that patients with high postoperative CRP level or low postoperative albumin level had shorter survival.

CRP/Alb ratio is the focus of this study. It reflected not only inflammation but also nutritional status of cancer patients. Previous studies have focused on whether pretreatment CRP/Alb ratio is a sensitive factor for predicting tumor prognosis and found consistent results: the elevated pretreatment CRP/Alb ratio was associated with a shorter OS in tumor patients [17–23]. As for the relationship between preoperative CRP/Alb ratio and long-term survival of patients with gastric cancer, we reached a conclusion consistent with previous studies. We also investigated whether preoperative CRP/Alb ratio is an independent predictor of DFS in patients with gastric cancer. In multivariate analysis, low CRP/Alb ratio predicted long DFS of gastric cancer patients (HR 1.42, 95% CI 1.08–1.87, P = 0.012). Moreover, we focused on the relationship between postoperative CRP/Alb ratio and prognosis of gastric cancer, which has rarely been reported. Arima K et al. retrospectively analyzed 142 patients with pancreatic cancer after surgery, and they found that the CRP/Alb ratio at the 14th day after surgery was correlated with patients' OS, and higher CRP/Alb ratio was associated with postoperative complications [33]. Similar to the above results, we found that high postoperative CRP/Alb ratio is associated with shorter OS (HR 2.03, 95% CI 1.55–2.66, P < 0.001) and shorter DFS (HR 1.72, 95% CI 1.29–2.30, P < 0.001) of gastric cancer. In summary, the results of our study suggest that postoperative CRP/Alb ratio is a novel independent risk factor for predicting prognosis of gastric cancer.

CRP/Alb ratio has been studied in other areas as well. Studies have shown that Nonalcoholic fatty liver disease (NAFLD) risk was associated with an increased risk of liver and most GI cancers, especially those of earlier onset [34]. One retrospectively study examined the relationship between the onset of NAFLD and clinicopathological findings to identify the risk factors associated with the development of NAFLD after gastrectomy. The incidence of postoperative NAFLD was 4.85%, and adjuvant chemotherapy with S-1 and high level of serum cholinesterase were considered as the risk factors for NAFLD occurring after gastrectomy for gastric cancer [35]. In addition, the malnutrition plays a central role to the development of NAFLD, likely leading to a reduced survival. Malnutrition splits in two directions; undernutrition and overnutrition. One of the hypotheses about the mechanisms inducing or worsening obesity and consequently NAFLD is the over-production of reactive oxygen species (ROS) [36]. In fact, albumin is the most abundant protein in human serum. One of the main roles assigned to albumin is as an indicator of malnutrition [37]. In other respects, studies have shown that T2DM is the precursor of NAFLD. Serum CRP/Alb ratio levels were significantly higher in complicated diabetic patients compared to controls [38].

To our knowledge, few studies have reported whether postoperative inflammatory factors can predict the long-term survival of gastric cancer. Thus, we incorporated both preoperative and postoperative CRP, albumin, and CRP/Alb ratio for analysis, in particular, postoperative CRP/Alb ratio has been studied more deeply. Eventually, we found that postoperative CRP/Alb ratio could act as an independent prognostic marker in early and locally advanced gastric cancer. Although the underlying mechanism is still unclear, researchers generally believe that there is systemic inflammatory response to occur in the presence of malignancy, stomach is one of the most important organs in the digestive system, and gastric cancer patients may suffer from malnutrition and inflammation after surgery. Therefore, postoperative CRP/Alb ratio is an independent risk factor for predicting the prognosis of gastric cancer.

To the best of our knowledge, this study is the first to elucidate the relationship between postoperative CRP/Alb ratio and the prognosis of early and locally advanced gastric cancer. It is suggested that in future clinical work, we should pay more attention to postoperative CRP/Alb in gastric cancer patients, and actively take measures to prevent infection and improve malnutrition in order to improve long-term survival of patients. However, there were several limitations. First, this is a retrospective, single-center study, and thus the accuracy of the results is not as high as that of multi-center prospective studies. Second, it did not carry out a study on the underlying mechanism in this study. If the corresponding in vitro and in vivo mechanism can be studied, the results will be more convincing. Third, different postoperative treatment and treatment duration may affect the outcome. So, our results should be validated by more cohort studies.

## Conclusion

As a simple and cheap inflammatory indicator, postoperative CRP/Alb ratio is an independent predictor of the prognosis of early and locally advanced gastric cancer. Detection of postoperative CRP/Alb ratio can help clinicians identify the high-risk patients, so as to make more reasonable intervention treatment.

## Data Availability

The datasets generated and/or analysed during the current study are not publicly available due [REASON WHY DATA ARE NOT PUBLIC] but are available from the corresponding author on reasonable request.
